# Pressure recording analytical method for cardiac output monitoring in children with congenital heart disease

**DOI:** 10.1186/cc9487

**Published:** 2011-03-11

**Authors:** Z Ricci

**Affiliations:** 1 Bambino Gesù Hospital, Rome, Italy

## Introduction

The Swan-Ganz catheter cannot be considered the gold standard in the pediatric setting for cardiac output (CO) monitoring, due to the unavailability of pulmonary artery catheters (PACs) of adequate size for children of all ages and weights and due to peculiar cardiovascular anatomies of some children with congenital heart disease (CHD). The pressure recording analytical method (PRAM) is designed for arterial pressure-derived continuous CO measurement and it does not need any starting calibration, central venous catheterization, or adjustments based on experimental data. The aim of this study was to validate PRAM in a cohort of children with CHD.

## Methods

An observational study was conducted on 25 children with CHD who underwent diagnostic cardiac catheterization (seven corrected tetralogy of Fallot, three corrected complete atrioventricular canal, 10 corrected transposition of great arteries and five dilative cardiomyopathy), aiming to compare CO measurement by PRAM and by PAC. Enrollment criteria were: biventricular anatomy in the absence of intracardiac shunts, weight <20 kg, prescheduled need for Swan-Ganz measurement of CO and arterial cannulation. The Swan-Ganz CO value considered in our study was the average measure deriving from three thermodilution boluses. The corresponding PRAM CO value was the average measure of those picked simultaneously with the three thermodilution boluses. All patients were anesthetized (2% inhaled sevoflurane and intravenous remifentanil at 0.1 μg/kg/minute) and mechanically ventilated.

## Results

The median patient age was 4 years (IQR 2.5 to 6) and median weight was 13 kg (IQR 9 to 17). A significant linear correlation between PRAM and Swan-Ganz measurements was found (*P *< 0.0001). In particular, Bland-Altman analysis showed a bias of 0.2 l/minute (SD 0.47) and 95% limits of agreement from -0.7 to 0.9 l/minute: the performance of this method seemed optimally coupled with PAC measurements when CO ranged from 1 to 2 l/minute whereas for higher COs the difference between the two methods increased. However, only three measurements fell out of the limits of agreement and all were at CO levels over 2.5 l/minute (more rarely observed in pediatric patients with CHD). Age, weight, heart rate and cardiac diagnosis were not significantly correlated with PRAM to Swan-Ganz difference.

## Conclusions

PRAM may be considered an accurate method in pediatric patients with CHD: these results should be validated in the pediatric ICU, also verifying PRAM's impact on clinical decision-making.

